# Postprandial Metabolome Following a Low Glycaemic Index Meal-Challenge Test: A Narrative Review

**DOI:** 10.21315/mjms2022.29.5.2

**Published:** 2022-10-28

**Authors:** Zhi Ch’ng Lau, Barakatun Nisak Mohd Yusof, Faridah Abas, Norasyikin Abd Wahab, Wan Zul Haikal Wan Zukiman, Amin Ismail

**Affiliations:** 1 Department of Dietetics, Faculty of Medicine and Health Sciences, Universiti Putra Malaysia, Selangor, Malaysia; 2 Research Centre of Excellent for Nutrition and Noncommunicable Diseases (NNCD), Faculty of Medicine and Health Sciences, Universiti Putra Malaysia, Selangor, Malaysia; 3 Department of Food Science, Faculty of Food Science and Technology, Universiti Putra Malaysia, Selangor, Malaysia; 4 Department of Medicine, Faculty of Medicine, Universiti Kebangsaan Malaysia, Selangor, Malaysia; 5 Department of Medicine, Faculty of Medicine and Health Sciences, Universiti Putra Malaysia, Selangor, Malaysia

**Keywords:** metabolomics, postprandial, glycaemic index, meal-challenge test, review

## Abstract

The Identifying the dynamic metabolome of the individual in response to a particular stimulus using a metabolomic approach is an emerging research area. Measuring the postprandial metabolite response utilising a meal-challenge test (MCT) provides information beyond the fasting state, which is especially important since human beings spend most of their time in the postprandial state. This is pertinent as an excessive rise in postprandial glycaemia is common in individuals with type 2 diabetes mellitus (T2DM), which puts them at a high risk of developing cardiovascular disease (CVD). While a low glycaemic index (GI) meal improves postprandial glycaemia and insulin levels in MCT studies among individuals with T2DM, its effect on metabolite changes in the postprandial state is unclear. This review summarises the perturbation in postprandial metabolites following a low GI meal in comparison to that following a usual or high GI meal and maps the metabolites in their metabolic pathways. We undertook a literature review using electronic databases, with the Medical Subject Headings (MeSH) terms, to retrieve relevant studies based on specific criteria. A total of seven related studies were documented. For the majority of metabolites studied, it was identified that metabolic regulation following an MCT extends beyond the glucose pathway. Altered metabolic pathways after the consumption of a low GI meal include: i) essential amino acid metabolism by altering the levels of plasma phenylalanine, tyrosine, lysine, leucine, isoleucine and valine; ii) glycolysis and tricarboxylic acid (TCA) metabolism by altering citrate and alanine, and iii) gut microbiota metabolism by altering betaine and acetate. The altered metabolites regulated the pancreatic insulin secretion and related to other dietary factors beyond GI modifications. These metabolomics data need to be interpreted cautiously because the metabolic changes analysed might not be due to the beneficial effects of a low GI meal. Validation of the putative metabolomic biomarkers following a dietary intervention MCT is suggested because researchers need to fully understand the kinetics and metabolism of individuals metabolite before reaching a solid conclusion. Further research characterising the metabotype based on habitual dietary patterns is warranted.

## Introduction

Metabolomics is a tool used to profile and quantify all low molecular-weight metabolites that are present in biological samples. This technique is instrumental in exploring the differences in the effects of particular stimuli on metabolic pathways in an organism. Different analytical platforms are utilised to unravel and quantify metabolite contents and then this information is combined with multivariate analysis tools, such as principal component analysis, for data interpretation and mining (1).

In recent years, interest has increased regarding applications of a metabolomics approach in nutrition research, examining metabolites following a meal-challenge test (MCT) (2). This approach is pertinent as most of the imbalances in diet-dependent metabolisms, such as inflammation and oxidation, are not detectable in the fasting state. The postprandial response of metabolites reflects the activation of endogenous metabolic pathways sensitive to food intake or absorbed macronutrients present in meals following consumption (3–6). Although extensive data in metabolites are available, the mapping and interpretation of observed differences in metabolites to the corresponding metabolic pathway remain challenging and warrant further validation. Furthermore, significant interindividual variations may be observed in response to an MCT due to the differences in gene polymorphisms, epigenetic patterns and gut microbiota compositions (7).

An excessive rise in postprandial glycaemia, defined as a 2-h post-meal glucose level of > 7.8 mmol/L (8), is a common feature of type 2 diabetes mellitus (T2DM) (9, 10). It has previously been observed that postprandial hyperglycaemia is more prevalent in Asians than Caucasians due to higher insulin resistance. In southeast Asia, the highest level of postprandial glycaemia was reported in Malaysia at 11 mmol/L (11). Furthermore, 40% of 1,077 Malaysian patients with T2DM had postprandial hyperglycaemia (12). Persistent and excessive elevations in the glycaemic level deserve necessary attention from healthcare professionals as such elevations lead to the development of atherosclerosis, thereby increasing the risk of cardiovascular disease (CVD) (13).

Low glycaemic index (GI) meals are commonly recommended to patients with T2DM to improve postprandial glycaemic and insulin responses (14, 15). GI ranks carbohydrates on a scale from 0 to 100 based on their effect to blood glucose after eating (16). For practical purposes, the GI concept can be categorised as low GI: ≤ 55, intermediate GI: 56–69 and high GI: ≥ 70. Foods with a high GI are rapidly digested, absorbed and metabolised, causing marked fluctuations in glycaemic levels, whereas foods with low GI produce a smaller fluctuation in glycaemic levels. Consuming an appropriate amount and selecting the right type of carbohydrate is beneficial in managing the postprandial glycaemic response in T2DM patients (17–20). Nonetheless, the effect of GI on metabolite changes in the postprandial state in individuals with T2DM is unclear.

Previous reviews on the metabolomics approach have focused on discovering dietary biomarkers in foods such as meat, fish, vegetables, citrus fruits, coffee and tea (6, 21, 22) with limited studies focusing on the meal GI. While reviews on metabolite markers among T2DM patients compared to those in their healthy counterparts have been documented, studies on the differences in metabolite markers after an MCT in the postprandial state are scanty (23–27). Thus, this review aimed to summarise the perturbation in postprandial metabolites following a low GI meal compared to those following a usual or high GI meal and map the metabolites into the corresponding metabolic pathways.

## Methods

A literature review was undertaken using electronic databases to retrieve relevant studies based on specific criteria. We utilised the Medical Subject Headings (MeSH) terms of ‘metabolomic’ AND ‘postprandial’ AND ‘NMR’. We restricted our search to studies related only to humans, published in English in the last 10 years. Studies that were included for review had the following study designs: randomised crossover or parallel clinical trials that applied a metabolomics approach to low GI MCT. Studies were excluded if they did not fulfil the selection criteria, were not applicable to research questions or were duplicate publications.

## Results

**Figure 1:**
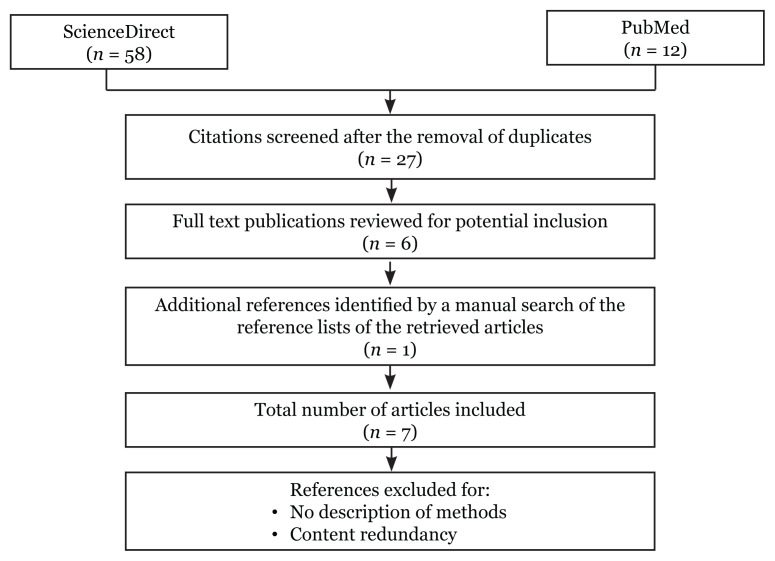
Flowchart of literature search

The flowchart of literature selection is presented in Figure 1. A total of seven related MCT studies were documented and summarised in Table 1. All studies were conducted among healthy individuals. The mean age was comparable within the retrieved studies except in this study (28), where they recruited post-menopausal women aged 61 (SD = 4.8) years old. Then, the mean body mass index (BMI) of individuals ranged from normal to slightly overweight. The majority of the studies had a randomised crossover design and utilised the nuclear magnetic resonance (NMR)-based metabolomics approach. There were five studies analysed the plasma samples while the remaining analysed the urine samples. When blood or urine samples were collected for metabolomics analysis, the time points ranged from 1 h to 8 h.

Since the purpose of this paper was to review recent research regarding low GI MCTs, we examined several studies that measured dietary GI. To the best of our knowledge, no MCT studies specifically investigated the effect of differing meal GI or GI values of food on the postprandial metabolomics profile. Therefore, we combined the studies that examined the effect of low GI food from different food groups such as rye (3, 28–31), wheat bran (32) and β-glucan fibre from barley (33). Then, we derived the GI values of the food items based on the International Table (34) and updated database on the GI website (Sydney University of Glycemic Index Research Services). There are three pathways and metabolites that are consistently reported to respond to low a GI meal: i) essential amino acid metabolism by altering the levels of plasma phenylalanine, tyrosine, lysine, leucine, isoleucine and valine; ii) glycolysis and tricarboxylic acid (TCA) metabolism by altering citrate and alanine; and iii) gut microbiota metabolism by altering betaine and acetate.

## Discussion

**Figure 2:**
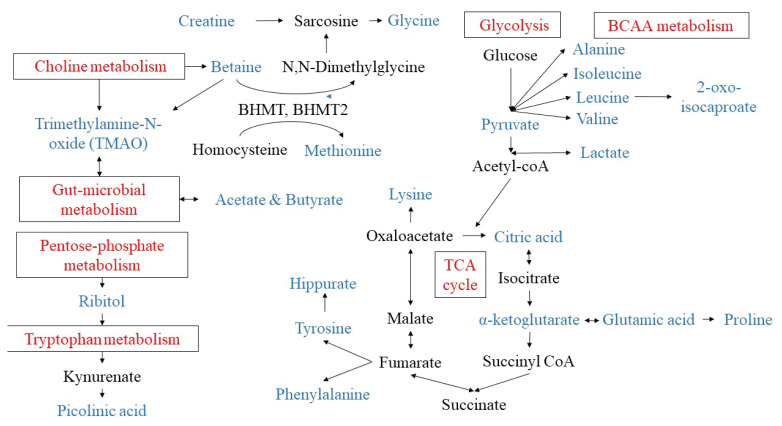
Mapping of perturbated postprandial metabolites following a low GI meal onto the metabolic pathway Notes: 
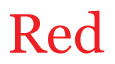
 = metabolic pathway; 
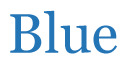
 = metabolites that were perturbated following low GI meal modification in MCT

This review paper seeks to summarise the available information regarding metabolomic biomarkers that can modulate the effects of low GI dietary intake concerning insulin resistance and T2DM. An attempt was made to map the metabolites perturbed following a low GI MCT onto the appropriate metabolic pathway, as shown in Figure 2. Surprisingly, the majority of the metabolites identified infer that metabolic regulation following an MCT extends beyond the glucose pathway.

First of all, postprandial differences in plasma metabolites related to essential amino acids metabolism such as phenylalanine, methionine, tyrosine, glutamic acid and lysine were identified after rye bread intake (3, 29, 30) and rye porridge (31). Branched-chain amino acids (BCAA) such as leucine, isoleucine and valine, and leucine catabolic intermediate (2-oxo-isocaproate) were also altered following a low GI MCT (28, 30, 31). These amino acids played a pivotal role in beta-cell function (5, 35) correlated with insulin resistance (36, 37) and have been independently associated with an increased risk of T2DM (23, 24, 35, 38). Moreover, a study by Würtz et al. (26) to determine the circulating metabolite predictors of glycaemia in middle-aged men and women concluded that alanine, phenylalanine and tyrosine are predictors of fasting and postprandial glucose levels at a follow-up of 6.5 years.

Consistently higher postprandial plasma phenylalanine and valine levels, lower isoleucine levels were observed after the consumption of low GI MCT (3, 28, 30, 40). More elevated phenylalanine and valine levels may promote insulin resistance and increased risk of T2DM (36, 38, 39) but it is also a biomarker of dietary protein intake. Lower plasma isoleucine levels reported agreed with Moazzami et al. (40), who studied the effect of an 8-week low GI meal in 33 healthy post-menopausal women. Surprisingly, lower isoleucine levels also reduce postprandial insulin demand despite similar glucose concentrations (28). Therefore, it is possible to hypothesise that insulin signalling in the skeletal muscle improved, increasing amino acids uptake in the circulation.

Secondly, the intake of low GI foods such as bran or rye resulted in a perturbation in glycolysis metabolism and the TCA cycle (e.g. alpha-ketoglutaric, pyruvic acid, citric acid, lactate and alanine), which were examined using a metabolomic approach (30, 32). Similarly, these metabolites were associated with insulin sensitivity and postprandial glucose upon follow-up at 6.5 years (26, 27, 36). A case-cohort study reported a significant positive association between plasma lactate and alanine levels with insulin resistance in 1 year (39). It was suggested that an elevated glycaemic level stimulated β-cells, led to the accumulation of glycolytic and TCA cycle intermediates during the first phase and even in the second phase of insulin secretion (41). However, the effect of low GI MCT in the regulation of plasma concentration or urinary excretion of these metabolites and its relation with the postprandial insulin response remains to be elucidated.

The metabolites derived from gut microbiota metabolism are altered after the consumption of low GI MCT. Low GI meal seems to modulate the products of colonic microbial fermentation by increasing the plasma short-chain fatty acids such as acetate and butyrate in a postprandial state, also decreasing the urinary formate formation after 6-month of low GI and high fibre intervention (31, 42). The gut microbiota metabolism of choline generated trimethylamine (TMA) and oxidised in the liver to produce trimethylamine-N-oxide (TMAO). Betaine is one of the precursors of TMAO and it is rich in rye bran. A 37% increase in plasma TMAO concentration was observed after 4-week consumption of a low GI diet (43). This finding supports the previous observation of lower urinary, higher plasma betaine and related metabolites (N-N-dimethylglycine) after low GI rye bread intake (32, 40). Overall, the changes in the metabolites following a low GI diet may benefit the gut microflora.

Despite this, the intake of different types of bread resulted in changes in metabolite levels involved in tryptophan metabolism (i.e. picolinic acid and ribitol) (3). This observation accords with a 40% increase in plasma kynurenate, the precursor of tryptophan metabolism, after a 4-week study of low glycaemic load (GL) and high fibre meal plan (43). A higher concentration of ribitol is reported to reduce hunger and food intake, whereas picolinic acid is an activator of the proinflammatory function of macrophages. This finding suggests that tryptophan metabolism may be protective against inflammation.

These studies indicated that several metabolite biomarkers can be detected by applying a metabolomic approach in nutritional intervention studies (i.e. modification with low GI meals) by analysing the metabolomic profiles derived from the comparison of inter and intra-individual dietary intakes. Furthermore, an MCT helps reveal postprandial metabolic responses that were not observed in a fasting condition. A low GI meal has been shown to reduce postprandial hyperglycaemia and the incremental benefit is similar to that offered by pharmacological agents such as alpha-glucosidase inhibitors that also target postprandial glycaemia. However, this review observed that the metabolites that underpin both therapies are not comparable. The metabolites induced by alpha-glucosidase inhibitor were 4-methylpyrogallol derivatives such as sulfate, methyl and glucuronide conjugates (44, 45).

These metabolomic results must be interpreted with a certain amount of caution because the altered metabolites may also be related to other dietary factors beyond GI modifications. Studies conducted by (29, 30) reported higher plasma and lower urinary creatine levels after consuming an egg and ham breakfast compared to that with a cereal breakfast. Creatine is mainly synthesised in the liver and is associated with protein consumption (29, 46). Similarly, higher citrate excretion may be due to increased citrus fruit intake, whereas a higher lysine content was found in eggs, meat and beans (29, 31). In addition, higher levels of the amino acid proline can be attributed to cheese or dairy product consumption (46) and higher methanol concentrations may be due to the higher pectin content of fruits and vegetables, which is metabolised by gut microflora (30).

The differences in the metabolites analysed from blood plasma or urine sample should be discussed. The urine sample contained predominantly low molecular weight metabolites, whereas blood plasma contained low and high molecular weight components such as lipoproteins (47). Metabolomics analysis of plasma samples provided measurement at a particular time point, whereas urine metabolites were the outcomes of metabolism (48). Another confounding factor that is considered is age as the physiological changes occur across different age groups, which influences the metabolite levels.

The existing body of literature regarding postprandial metabolic perturbation following low GI meal modification has dealt with healthy individuals. However, little attention has been paid to the metabolic effects of low GI meals on patients with T2DM. It has been reported that there are differences in metabolites including carbohydrate, amino acids and choline-containing phospholipids between healthy individuals and individuals with glucose dysmetabolism such as impaired fasting glucose and T2DM (23, 24). Questions about whether the metabolite biomarkers induced are similar among healthy individuals and patients with T2DM remain unanswered.

In summary, the findings provide additional insight into the postprandial metabolic response following a low GI meal. However, the generalisability of these biomarkers is subject to certain limitations. Validation of putative metabolic biomarkers is needed following a dietary intervention MCT, applying a metabolomic approach within several study design conditions and sampling strategies. Additionally, a lipidomic platform should be applied in future research to establish a postprandial lipidome profile following low GI meal consumption.

## Conclusion

Overall, this review provided evidence for the perturbation of several postprandial metabolites after consuming a low GI meal, and it discussed these metabolites concerning the pathophysiology of T2DM. The metabolites induced in response to a low GI meal regulated pancreatic insulin secretion but further validation is warranted. Advances in metabolomics research allow for the comprehensive identification of metabolites, aiding in understanding the effects of particular stimuli on metabolic pathways; thus, it is possible to understand the subtle effects of diet on human metabolic status. In the future, research should focus on applying a metabolomic or lipidomics approach for characterising biomarker profiles that reflect habitual dietary patterns, also known as the metabotype. More extensive metabolomic analysis should be performed, targeting specific populations such as in T2DM patients, and in individuals with circadian disruption. More tremendous efforts in this field are required to improve the health status of humans in the future.

## Figures and Tables

**Table 1:** MCT conducted to elucidate the perturbations in postprandial metabolites following a low GI meal

Subjects, mean age (SD) (years old)	BMI mean (SD) (kg/m^2^)	Study design and technique	Time-points (hours)	Food	Functional concept (GI value)	Plasma metabolite after low GI MCT	Urine metabolite after low GI MCT	Reference
13 women, 3 men 23.0 (3.7)	22.0 (1.8)	Randomised controlled trial GC-MS	1	Sourdough fermented endosperm rye bread versus White wheat bread	48 versus 65	↑ Phenylalanine ↑ Methionine ↓ Tyrosine ↓ Glutamic acid ↑ Alpha-ketoglutaric ↓ Pyruvic acid ↑ Citric acid ↓ Picolinic acid ↑ Ribitol	-	Bondia-Pons et al. (3)
19 women 61.0 (4.8)	26.0 (2.5)	Randomised crossover trial NMR + LC-MS	3	Wholemeal rye bread versus Refined wheat bead versus Refined rye bread	54 versus 65 versus 78	↓ Leucine ↓ Isoleucine ↓ 2-oxo-isocaproate		Moazzami et al. (28)
7 women, 7 men 27.8 (6.5)	22.7 (2.6)	Randomised crossover trial NMR	2	Wheat bran or aleurone versus control (food not mentioned)	43	-	↑ Lactate ↑ Alanine ↑ N-acetylaspartate (NAA) acid ↑ N-acetylaspartylglutamate (NAAG) ↑ Citrate ↑ Hippurate ↑ 2-hydroxyisobutyrate ↓ Betaine	Garg et al. (32)
10 women, 10 men 23–60	21–33	Randomised crossover trial NMR + GC-MS	8	Rye porridge with different inulin: gluten ratios versus Plain wholegrain rye porridge versus Refined wheat bread	29 versus 60 versus 65	↑ Essential amino acids ↑ Short chain fatty acids (acetate and butyrate)	-	Shi et al. (31)
12 women, 12 men Female: 24.4 (8.2) Male: 27.3 (11.2)	Female: 23.1 (2.3) Male: 22.8 (2.1)	Randomised crossover trial NMR	3	Cereal breakfast: orange juice, oat puffs with milk and a rye bread sandwich with hard cheese and fresh tomato versus Egg and ham breakfast: Orange juice, scrambled eggs, white beans in tomato sauce, fried pork loin, tomato and toasted white bread with orange marmalade	50 versus 73	-	↓ Creatine ↓ Citrate ↓ Lysine ↑ Erythrose	Rådjursöga et al. (29)
12 women, 12 men Female: 24.4 (8.2) Male: 27.3 (11.2)	Female: 23.1 (2.3) Male: 22.8 (2.1)	Randomised crossover trial NMR	3	Cereal breakfast versus Egg and ham breakfast	50 versus 73	↑ Proline ↑ Tyrosine ↑ N-acetylated amino acids ↑ Valine ↑ 3-hydroxybutyrate ↓ Alanine ↓ Creatine ↓ Methanol ↓ Isoleucine ↓ Glycine	-	Rådjursöga et al. (30)
8 women, 6 men 22.9 (2.1)	19–27	Randomised crossover trial NMR	4	3.3g mixed linkage β-glucan oat versus Mutant barley versus Mother barley β-glucan versus Control without fibre	57 versus 62 versus 50 versus 65	No systematic differences in the lipoprotein subclasses were found	-	Mikkelsen et al. (33)

Notes: GC-MS = gas chromatography-mass spectrometry; LC-MS = liquid chromatography-mass spectrometry; NMR = nuclear magnetic resonance
